# Diagnostic efficacy and safety of radial probe endobronchial ultrasound-guided transbronchial needle aspiration for adjacent lesions in segmental or subsegmental bronchi: a single-center retrospective study

**DOI:** 10.1186/s12890-023-02781-1

**Published:** 2023-12-04

**Authors:** Debin Ma, Junli Zhang, Qingwei Zeng, Baining Li, Meili Gong, Zhiyuan Zhang, Zhuang Ma

**Affiliations:** Department of Respiratory and Critical Care Medicine, General Hospital of Northern Theater Command, Shenyang, China

**Keywords:** Radial probe endobronchial ultrasound (REBUS), Convex probe endobronchial ultrasound (CEBUS), Transbronchial needle aspiration (TBNA), Segmental bronchi, Diagnostic yield

## Abstract

**Background:**

Peripheral lung lesions can be sampled using various techniques, including computer tomography-guided transthoracic needle aspiration, electromagnetic navigation bronchoscopy, virtual navigation bronchoscopy, and radial probe endobronchial ultrasound transbronchial lung biopsy. Mediastinal lesions can be sampled using techniques like convex probe endobronchial ultrasound-guided transbronchial needle aspiration (CEBUS-TBNA) and endoscopic ultrasound-fine-needle aspiration. However, effective, safe techniques for lesions adjacent to the segmental or subsegmental bronchi are lacking. Herein, we retrospectively evaluated the diagnostic yield and safety of radial probe endobronchial ultrasound-assisted transbronchial needle aspiration (REBUS-TBNA) for lesions adjacent to the segmental bronchi, and explored the factors related to diagnostic yield.

**Methods:**

We retrospectively analyzed the diagnostic yield and safety of REBUS-TBNA cases performed in our department from January 2019 to December 2022. Observation group patients had undergone REBUS-TBNA for lesions adjacent to the segmental bronchi; control group patients had undergone CEBUS-TBNA for mediastinal or hilar lesions. Patient characteristics and lesion sizes, diagnostic yield, adverse events, and relations between diagnostic yield and clinical characteristics were analyzed.

**Results:**

There were not statistically significant between-group differences in sex, age, diagnostic yield, or rate of adverse events. The observation group (n = 25; 17 male, 8 female) had a mean age of 64.76 ± 10.75 years. The average lesion size was 4.66 ± 1.07 cm, and lesions were predominantly in the upper lobes (80%). REBUS-TBNA diagnostic yield was 84%, with no adverse events reported. Diagnostic yield was not associated with lesion size or extent of bronchial stenosis; however, it was positively correlated with number of punctures. Patients with > 3 punctures had a significantly higher diagnostic yield than those with ≤ 3 punctures.

**Conclusions:**

REBUS-TBNA is a safe, effective diagnostic technique, particularly for lesions adjacent to the segmental or subsegmental bronchi of the upper lobe. Performing more than three punctures during the procedure improves the diagnostic yield. Larger-scale studies are warranted to confirm these results, and to further explore the clinical value of REBUS-TBNA.

## Background

The COVID-19 pandemic increased the prevalence of lung computed tomography (CT) examinations [1, 2]. The rate of detecting lung nodules, pulmonary masses, and mediastinal masses has also increased significantly. Techniques like non-invasive liquid biopsy test, computer tomography-guided transthoracic needle aspiration (CT-TTNA), electromagnetic navigation bronchoscopy (ENB), virtual navigation bronchoscopy (VNB), and radial probe endobronchial ultrasound-assisted transbronchial lung biopsy (REBUS-TBLB), are used to sample peripheral lung tissue lesions [3–9]. For mediastinal lesions, techniques like convex probe endobronchial ultrasound-guided transbronchial needle aspiration (CEBUS-TBNA) and endoscopic ultrasound-fine-needle aspiration are used [10–14]. However, for lesions located in the segmental or subsegmental bronchi from the 3rd − 5th order (i.e., not peripheral or mediastinal regions), CEBUS and endoscopic ultrasound (EUS) are usually inadequate. CT-TTNA is high-risk, with complication rates up to 40% and pneumothorax rates of 25%, and carries a significantly higher occurrence of pneumothorax in lesions in the distal chest wall compared with those in the proximal chest wall [4–7]. Since these lesions typically do not involve the airway mucosa, TBLB and brushing techniques are also unsuitable. Therefore, effective, safe techniques are urgently needed for the diagnosis of segmental or subsegmental bronchi-adjacent lesions.

EBUS is a bronchoscopic examination technique that utilizes ultrasound to visualize the airway wall, lung, and mediastinal structures. It includes convex probe EBUS (CEBUS) and radial probe EBUS (REBUS).

CEBUS-TBNA is a cutting-edge technology that has emerged in the last two decades as a highly effective diagnostic tool. Specifically, it excels in identifying enlarged mediastinal and hilar lymph nodes (LNs) in patients exhibiting benign or malignant conditions through CT or positron emission tomography-CT (PET-CT). Numerous clinical studies have attested to its efficacy, noting that it is both cost-effective and safe in procuring diagnostic specimens. In fact, its diagnostic yield is often comparable to, or even surpasses, that of surgical mediastinoscopy. The applications of CEBUS-TBNA are vast and include diagnosing intrapulmonary tumors, determining LN staging in lung cancer patients, uncovering unexplained hilar and/or mediastinal LN enlargement, and pinpointing mediastinal tumors [10–14].

Contrastingly, there is a dearth of published studies focusing on radial probe EBUS-assisted transbronchial needle aspiration (REBUS-TBNA) [15, 16]. On the other hand, a plethora of research has been conducted on REBUS-TBLB, [17–21]. REBUS technique involves the insertion of an independent ultrasound probe into the bronchial lumen through the working channel of the bronchoscope. This device is characterized by non-embedded, non-real-time monitoring, and circular scanning capabilities. While REBUS-TBNA enables access to more distant and finer bronchi, its primary limitation lies in the inability to provide real-time monitoring during the puncture procedure. Table 1 comprehensively outlines the main differences between CEBUS-TBNA and REBUS-TBNA techniques.

**Table 1:** CEBUS-TBNA vs. REBUS-TBNA comparison

	CEBUS-TBNA	REBUS-TBNA
Flexibility of bronchoscope for view	60°	360°
Real time TBNA potential	Yes	No
Doppler mode potential	Yes	No
Ultrasound probe frequency*	5–12 MHz	20 MHz
Tissue Penetration	Low	High

*Higher frequency leads to better resolution and image quality and less penetration; low-frequency leads to better penetration but lower resolution (i.e., lower quality) images

For segmental or subsegmental bronchi-adjacent lesions that are inaccessible via CEBUS-TBNA but can be reached by flexible bronchoscopy, REBUS-TBNA plays an important role [15, 16]. However, there are currently limited reports on the diagnostic efficacy and safety of this technique. Thus, in this retrospective study, we aimed to evaluate the diagnostic efficacy and safety of REBUS-TBNA for segmental and subsegmental bronchi-adjacent lesions, and to explore factors that may affect diagnostic yield.

## Methods

### Patients

This retrospective study included patients who underwent REBUS-TBNA and CEBUS-TBNA in the Department of Respiratory and Critical Care Medicine at our hospital between January 1, 2019, and December 31, 2022. The patients included in the observational group were those who had undergone REBUS-TBNA to access lesions adjacent to segmental or subsegmental bronchi that were deemed unreachable by CEBUS-TBNA. Typically, various factors such as the device status, consumables batch, operator, and gender can potentially influence study results. To minimize confounding factors, the control group has established inclusion criteria that require enrolling the first or second CEBUS-TBNA patient (due to incomplete or missing medical records of the first patient) with the same operator and gender following every REBUS-TBNA patient. The exclusion criterion is that patients with incomplete or missing medical records are not eligible for the study. Included patients’ final diagnosis was based on cytology, histopathology, microbiology examination, imaging findings, and clinical features.

### Equipment

The following equipment, all produced by Olympus Corporation, Tokyo, Japan, was used: bronchoscope (BF-1T260), flexible bronchoscope (BF-260), EBUS bronchoscope (BF-UC260FW), bronchoscope main unit (BF-CLV-260), intracavitary ultrasound main unit (EndoEcho EU-ME2), intracavitary ultrasound probe (UM-S20-17 S), and 22G WANG TBNA needle (NA-201SX-4022).

### Protocol

Prior to the procedure, preoperative discussions were held, and the operating physician reviewed the patient’s chest CT images to determine legion size(s) and location(s). Preoperative preparations followed routine procedures for bronchoscopy, including a six-hour fasting period. Before the procedure, lidocaine nebulization and topical anesthesia with lidocaine jelly were administered to the pharynx, and the patient received continuous oxygen supplementation during the examination, while vital signs were monitored.

#### REBUS-TBNA

A flexible bronchoscope was inserted through the nose, and the target location was explored to observe the extent of bronchial stenosis and whether the mucosa was involved. Then, the radial probe ultrasound probe was passed through the working channel of the bronchoscope into the bronchial lumen, and a detailed examination was performed to determine the optimal puncture location and depth. The flexible bronchoscope was fixed, and the radial probe ultrasound probe was withdrawn. A puncture needle was then inserted and adjusted to the previously determined optimal puncture location and depth. After entering the lesion, the inner stylet was withdrawn, and a negative pressure syringe was connected. The puncture needle was moved back and forth 20 times without X-ray fluoroscopy. The number of needle passes for each lesion ranged from 1 to 5, depending on factors such as patient tolerance, bleeding, and specimen quality. Obtained specimens were used for histopathological examination, liquid-based cytology, microbiology examination, and, as needed, immunohistochemistry, special staining, and pathogen metagenomic next-generation sequencing (mNGS) tests.

#### CEBUS-TBNA

A regular bronchoscope was inserted through the nose to observe the mucosa of the bronchial lumen in detail. Then, an ultrasound bronchoscope was inserted through the mouth, and the convex probe ultrasound probe was advanced near the lesion. Under real-time ultrasound guidance, the probe was brought close to the airway wall, and the distal balloon was inflated with 0.5–1 ml of normal saline to fill the bronchus. Lymph nodes were identified and measured, and the optimal puncture point was determined to avoid puncturing large blood vessels. A puncture needle was then inserted through the working channel of the bronchoscope. After entering the lesion, the inner stylet was withdrawn, and a negative pressure syringe was connected. The puncture needle was moved back and forth 20 times. The number of needle passes for each lesion ranged from 1 to 5. Obtained specimens were used for histopathological examination, liquid-based cytology, microbiology examination, and, as needed, immunohistochemistry, special staining, and pathogen mNGS tests.

Patients who received a definitive diagnosis via EBUS-TBNA (e.g., tumor, tuberculosis, sarcoidosis, fungal infection) were defined as positive. Patients who could not receive a definitive diagnosis were defined as negative.

In case of post-procedure bleeding, diluted epinephrine or cold saline was used for local hemostasis. After confirming no active bleeding, the procedure was completed. Postoperative adverse events, such as worsening cough, chest pain, pneumothorax, bleeding/hemoptysis requiring clinical intervention, new-onset/worsening hypoxemia, and death, were recorded.

### Statistical analysis

Data were analyzed using SPSS Statistics version 23 (IBM, Armonk, NY, USA). Continuous variables are expressed as mean ± standard deviation, and categorical variables are presented as frequencies and percentages. Independent-samples *t-*tests were used to compare between-group age distributions. Fisher’s exact probability test was used to compare between-groups diagnostic yield and incidence of adverse events. Clinical characteristics for patients with different diagnoses (“positive” or “negative”) were compared using fisher’s exact probability test. A p-value < 0.05 was considered statistically significant.

## Results

### Patient characteristics

A total of 25 patients were included in the observational group (Table 2). Among them, 17 were male and 8 were female, with a mean age of 64.76 ± 10.75 years. The control group (Table 3) included 25 patients, with 17 males and 8 females, and a mean age of 60.44 ± 9.56 years. There were not statistically significant gender or age differences between the groups (p > 0.05).

**Table 2:** Clinical characteristics of REBUS-TBNA patients

No.	Sex	Age	Lesion diameter (cm)	Lesion location	Extent	No. punctures	Pathology	Diagnosis	Adverse events
1	F	73	6.2	RML B4	Mild	4	Inflammation	P	NO
2	F	62	2.8	LUL B4	Mild	4	Inflammation	P	NO
3	F	72	4.3	LUL B4b	Mild	4	Adenocarcinoma	P	NO
4	F	68	2.4	LUL B4	Mild	2	Blood	N	NO
5	M	82	3.7	RUL B3	Atretic	5	Squamous Carcinoma	P	NO
6	F	69	4.1	RUL B2	Mild	5	Adenocarcinoma	P	NO
7	M	64	5	RUL B3	Mild	5	Inflammation	P	NO
8	F	59	4.9	LUL B4	Atretic	3	ND	N	NO
9	M	64	3.8	LUL B1 + 2	Mild	4	SCLC	P	NO
10	M	77	5.3	RML B5	Mild	4	Adenocarcinoma	P	NO
11	M	70	3.8	RUL B3	Mild	5	Adenocarcinoma	P	NO
12	M	77	6.1	RML B4	Mild	3	Blood	N	NO
13	M	69	4.5	RUL B2	Atretic	5	Granuloma	P	NO
14	M	56	4.7	RUL B3	Atretic	5	Granuloma	P	NO
15	M	78	5.2	RLL B10	Mild	3	ND	N	NO
16	F	60	6	RUL B2	Mild	5	SCLC	P	NO
17	M	55	4.8	LUL B1 + 2	Mild	4	SCLC	P	NO
18	M	29	3	LUL B1 + 2	Mild	5	Granuloma	P	NO
19	M	62	4.1	RUL B2	Mild	4	Adenocarcinoma	P	NO
20	M	55	5.2	RUL B3	Mild	5	Adenocarcinoma	P	NO
21	F	62	4.6	RUL B2	Atretic	4	SCLC	P	NO
22	M	67	5.6	RUL B3	Mild	5	Adenocarcinoma	P	NO
23	M	53	4.3	LLL B10	Mild	5	Adenocarcinoma	P	NO
24	M	65	6.7	RUL B2	Mild	5	Adenocarcinoma	P	NO
25	M	71	5.3	RUL B3	Mild	5	SCLC	P	NO

M: male; F: female; RUL: right upper lobe; RML: right middle lobe; RLL: right lower lobe; LUL: left upper lobe; LLL: left lower lobe; Extent: extent of bronchial stenosis; Atretic: bronchi is atretic; SCLC: small cell lung cancer; ND: non-diagnostic; P: positive diagnosis; N: negative diagnosis

**Table 3:** Clinical characteristics of CEBUS-TBNA patients

No.	Sex	Age	Lesion location	Pathology	Diagnosis	Adverse events
1	F	71	#4R, mass	Adenocarcinoma	P	NO
2	F	62	#4R, mass	Small cell lung cancer	P	NO
3	F	67	#7, #11R, mass	Granuloma	P	NO
4	F	52	#7, mass	Granuloma	P	NO
5	M	78	#4R, #7	Small cell lung cancer	P	NO
6	F	51	#7, #11R	Granuloma	P	NO
7	M	65	#4R, #7, #11R	Adenocarcinoma	P	NO
8	F	44	mass	Granuloma	P	NO
9	M	57	mass	Squamous Carcinoma	P	NO
10	M	58	#7	Adenocarcinoma	P	NO
11	M	56	#7, mass	Adenocarcinoma	P	NO
12	M	59	#7, #11L	Adenocarcinoma	P	NO
13	M	62	#4R, #7, #4L	Small cell lung cancer	P	NO
14	M	58	#4R, mass	Small cell lung cancer	P	NO
15	M	75	#11R	Inflammation	P	NO
16	F	50	#4R, mass	Granuloma	P	NO
17	M	51	#4R, mass	Adenocarcinoma	P	NO
18	M	73	#7, #11R, mass	Inflammation	P	Transient
19	M	56	#7, mass	Adenocarcinoma	P	NO
20	M	71	#4R, #7	Granuloma	P	NO
21	F	49	#7, #11R	ND	N	NO
22	M	48	#4R, #7, #11R	ND	N	NO
23	M	72	mass	Adenocarcinoma	P	NO
24	M	56	mass	Inflammation	P	NO
25	M	70	#7	Granuloma	P	NO

M: male; F: female; ND: non-diagnostic; P: positive diagnosis; N: negative diagnosis; Transient: transient worsening cough

In the observational group, the average lesion size was 4.66 ± 1.07 cm, and they were mainly located in the upper lobes of both lungs, with 7 cases (28%) in the left upper lobe, 13 cases (52%) in the right upper lobe, 3 cases (12%) in the right middle lobe, and 1 case (4%) each in the right and left lower lobes. All lesions were inaccessible via CEBUS-TBNA due to anatomical structures or severe bronchial stenosis. The extent of bronchial stenosis was mainly mild (20 cases, 80%), with a small number of cases showing complete external occlusion (5 cases, 20%). The median number of punctures was 4 times (note that performing as many punctures as possible to obtain sufficient specimens was recommended). The 5 patients who underwent fewer than the median number of punctures were due to poor patient tolerance, difficulty with lesion puncturing, and bleeding risk. Pathology results included tumors (15 cases, 60%), inflammation (3 cases, 12%), granulomas (3 cases, 12%), blood clots (2 cases, 8%), and non-diagnostic samples (2 cases, 8%). Patients with blood clots (2 cases) and non-diagnostic samples (2 cases), combined clinical features and other tests, were ultimately not diagnosed.

In the control group, all lesions were located in the mediastinum or hilum. Pathology results consisted of tumors (13 cases, 52%), granulomas (7 cases, 28%), inflammation (3 cases, 12%), and non-diagnostic samples (2 cases, 8%). The patients with non-diagnostic samples (2 cases), combined clinical features and other tests, were ultimately not diagnosed.

### Diagnostic yield and incidence of adverse events

In the observational group (Table 4), there were 21 confirmed cases and 4 undiagnosed cases, resulting in a diagnostic yield of 84%. In the control group (Table 4), there were 23 confirmed cases and 2 undiagnosed cases, resulting in a diagnostic yield of 92%. There was not a statistically significant between-group difference in the diagnostic yield (p > 0.05).

The incidence of adverse events in the observational group (Table 4) was 0. In the control group (Table 4), the incidence of adverse events was 4%, with 1 patient experiencing mild exacerbation of cough within 24 h, which resolved quickly without intervention. There was not a statistically significant between-group difference in the incidence of adverse events (p > 0.05).

No other complications occurred during the procedures in either group, except for minor bleeding at the puncture site. Nor were there any postoperative cases of chest pain, pneumothorax, clinically significant bleeding/hemoptysis, new-onset or worsened hypoxemia, or deaths in either group.

**Table 4:** Diagnostic yield and incidence of adverse events

Group	Positive	Negative	Total	P value
Diagnosis				
REBUS-TBNA	21	4	25 (84%)	0.667
CEBUS-TBNA	23	2	25 (92%)	
Adverse events				
REBUS-TBNA	0	25	25 (0)	1
CEBUS-TBNA	1	24	25 (4%)	

### Relations between diagnostic yield and clinical characteristics

The diagnostic yield of REBUS-TBNA (Table 5) was unassociated with lesion size or the extent of bronchial stenosis (p > 0.05). However, there was significant, positive correlation with number of punctures, with patients who underwent > 3 punctures having a significantly higher diagnostic yield than those who underwent ≤ 3 punctures (p < 0.05).

**Table 5:** Relations between diagnostic yield and clinical characteristics

Group	Positive	Negative	Total	P value
Lesion diameter				
≥ 4.7 cm	10	3	13 (76.9%)	0.593
< 4.7 cm	11	1	12 (91.7%)	
Times				
≤ 3	0	3	3 (0)	0.002
> 3	21	1	22 (90%)	
Extent				
Mild	17	3	20 (70%)	1
Atretic	4	1	5 (80%)	

Positive: positive diagnosis; Negative: negative diagnosis; Times: number of punctures; Extent: extent of bronchial stenosis; Atretic: bronchi is atretic

## Discussion

### REBUS-TBNA access to adjacent lesions of the upper lobe segmental and subsegmental bronchi

CEBUS-TBNA is a widely used efficacious, minimally invasive technique for staging lung cancer and obtaining samples from mediastinal and hilar lymph nodes/masses. In addition to its uses in malignant diseases, it is also the preferred diagnostic method for non-malignant conditions such as tuberculosis, sarcoidosis, and fungal infections [22–24]. However, CEBUS-TBNA limitations (Tables 1 and 6) include sampling lesions in the upper lobe, due to anatomical structures and angles [25, 26].

Techniques including CT-TTNA, ENB-TBLB, VNB-TBLB, and REBUS-TBLB have been used to sample peripheral lesions of the upper lobe. However, sampling lesions adjacent to the segmental or subsegmental bronchi of the upper lobe remains challenging. CEBUS-TBNA is often unable to reach these locations, CT-TTNA has a high complication rate, and techniques like TBLB and brush biopsy are not ideal because these lesions typically do not involve the airway mucosa. In contrast, REBUS-TBNA is an effective, safe method that overcomes these limitations and can be used to sample lesions adjacent to the segmental or subsegmental bronchi of the upper lobe.

### REBUS-TBNA and CEBUS-TBNA diagnostic yields and adverse events

Although REBUS-TBNA and CEBUS-TBNA both have advantages, limitations, and unique indications (Table 6), comparisons between them are indirect. To evaluate the diagnostic yield and safety of REBUS-TBNA more effectively, within a clinical context, we included a control group of patients who underwent CEBUS-TBNA. To minimize confounding factors (e.g., equipment operation status, consumable batch, operator experience, gender), these patients were gender-matched and each was the next CEBUS-TBNA patient of the same operator who performed the observational patient’s REBUS-TBNA.

**Table 6:** Advantages and limitations of REBUS-TBNA vs. CEBUS-TBNA

	REBUS-TBNA	CEBUS-TBNA
Advantages	1. Large bendable angle 2. Reach more distant and finer bronchi	1. Real-time monitoring 2. Doppler mode
Limitations	non-real-time monitoring	Poor accessibility of upper lobe lesions or distal lesions
Unique indications	1. Lesions in segmental or subsegmental bronchi 2. Peripheral pulmonary lesions	Mediastinal or hilar lesions

CEBUS-TBNA is widely accepted as an efficacious, safe technique, which plays a crucial role in the diagnosis and treatment of various benign and malignant lung diseases [27, 28]. For diagnosing and staging lung tumors, CEBUS-TBNA demonstrates high sensitivity and diagnostic yield, and low complication rates [29, 30]. The American College of Chest Physicians non-small cell lung cancer guidelines report a sensitivity of 91% for diagnostic EBUS-TBNA [24]. Several studies have reported complication rates up to 1.44% [30–34]. Substantial evidence also suggests that CEBUS-TBNA exhibits high diagnostic yield and low complication rates in diagnosing benign diseases [35, 36]. These CEBUS-TBNA findings align with the diagnostic yield of 92% and complication rate of 4% reported herein.

Concerns have been raised regarding the lower diagnostic yield and inferior safety profile of REBUS-TBNA as a non-real-time guided technique. However, our finding of non-significant differences in diagnostic yield and adverse events between it and CEBUS-TBNA indicates that they share these characteristics. This establishes REBUS-TBNA as a safe, effective diagnostic technique, particularly for lesions adjacent to the segmental or subsegmental bronchi of the upper lobe.

### Puncture frequency improves diagnostic yield

Factors like lesion location, operation angle, patient tolerance, specimen quality, and operator experience can influence the number of punctures during a procedure. Our retrospective study revealed that performing more than three punctures results in a significantly higher diagnostic yield. Thus, we recommend performing at least four punctures with REBUS-TBNA, when conditions allow, and patient safety is ensured.

### Limitations

This retrospective study was not without limitations. First, it was a small-sample, single-center, retrospective study. There are few related reports, and only two similar studies, to which to compare our REBUS-TBNA diagnosis rate of 84% and adverse event rate of 0. Huang et al. [16] reported diagnosis and adverse event rates of 93.75% (15/16) and 12.5% (2/16), respectively, consistent with our findings. Song et al.’s [15] diagnosis rate was 42.1% (8/19), differing slightly from our report. The reasons for these discrepancies (e.g., sample sizes, lesion sizes, lesion locations) require clarification using large, multi-center samples. Second, REBUS-TBNA lacks a mature technologic comparison with the same indications. However, comparing with CEBUS-TBNA, widely recognized for its high diagnostic rate and low adverse event incidence, we indirectly illustrated that REBUS-TBNA has similar characteristics. Third, a lack of long-term follow-up disallowed calculation of broader sensitivity. Based on these cumulative findings, continuing to explore the clinical value of REBUS-TBNA is warranted.

## Conclusions

REBUS-TBNA is not only a safe and effective diagnostic technique, but it also opens up new possibilities in the field of pulmonary lesion diagnosis. Specifically, REBUS-TBNA has demonstrated unique advantages for lesions located in the segmental or subsegmental bronchi of the upper lobe. Furthermore, the value of guided sheath (GS)-TBNA in needle aspiration biopsy for peripheral pulmonary lesions (PPLs) located far from the central airway is gradually being recognized [37–39]. Looking ahead, it is foreseeable that REBUS-GS-TBNA holds great potential in the diagnosis of pulmonary lesions. We will continue to forge ahead, actively exploring and enhancing the clinical value of REBUS-TBNA, in order to bring more precise and efficient diagnostic tools to the medical community and patients alike.

## Data Availability

All data generated or analyzed during this study are included in this article. Further inquiries can be directed to the corresponding author.
